# The Board of Examiners and the Atlanta Clinic

**Published:** 1896-12

**Authors:** E. R. Anthony

**Affiliations:** President of Board of Medical Examiners; Griffin, Ga.


### CORRESPONDENCE.

### THE BOARD OF EXAMINERS AND THE ATLANTA CLINIC.

GRIFFIN, GA., October 27, 1896.

*To the Editor of the Atlanta Medical and Surgical Journal:*

My attention has just been called to a very caustic and unjust criticism of the Board of Medical Examiners of Georgia, published as an editorial in the October number of the *Atlanta Clinic.* Please allow me space in your columns to reply to the same.

I suppose the public acts of all public officers are liable to be criticised, and self-appointed critics, like the poor, we have with us always. This spirit of censoriousness nearly always has its origin in ill-nature, and as Lady Blessington says, "They consider the severity of their censures on the supposed failings of others as an atonement for their own." There are two things which frequently neutralize the sting of a criticism; the obscurity of the source and the injustice of the criticism.

The board is called to task by the writer of the article, and is accused of being derelict of its duty in that it does not prosecute quacks and rid the State of their presence. This same writer says, "The law is very plain." Yes, indeed, it is plain, but not plain enough it seems for this brilliant writer to understand it. The end of the law under which the board was created was to protect the people against irresponsible and incompetent practitioners of medicine by requiring that no one be allowed to practice medicine (since 1895—the law is not retroactive) without having passed a satisfactory examination before the board. The function of the board, then, is to examine eligible applicants and to pass on the competency of those examined to become practicing physicians. There its function ceases. The object of the board, then, is not to prosecute quacks, nor usurp the functions of the courts. Nor, indeed, is it to determine whether those who pass shall henceforth conform to professional ethics, nor does it guarantee that every one who passes shall become an efficient physician. What the board has to do is to determine whether applicants have requisite knoweledge—only this. Its function, then, is constructive immediately, destructive remotely. It must destroy gradually the evils of ignorance which have cursed the profession, by seeing that no man enters it whose knowledge is not sufficient to qualify him to practice. The board is in a measure responsible for the knowledge of those who pass, but by no means for their ability to apply what they know. Our critic can find no sensible lawyer in this State who will construe the law in such a way as to make it the duty of the board to prosecute any violators of the law. The law does make provision for the punishment of its violators, but no such thing as making the board a board of prosecuting officers is contemplated by the law. "The law upon the subject is plain, your duty is a plain one," says our critic. Our duty *is* a plain one. That duty is simply for the board to meet twice yearly and examine such applicants for license to practice medicine as are eligible for examination, and pass upon their qualifications. If the board should see fit to meet oftener it may do so. We had five sessions last year. At every meeting, I am sure each member conscientiously performed his duty as he understood it. And we have given an account of our stewardship to the proper person. An official statement of the work done by the board is in the hands of the Governor.

While it is not the legal duty of the board to prosecute quacks or violators of this law, we admit that there is a moral obligation resting upon us as physicians and as good citizens to do what we can to bring offenders against this law to punishment. But this moral obligation is no more binding upon us than it is upon any other reputable physician or good citizen of this State. It is the duty of all good citizens, of all reputable physicians especially, to report to the proper authorities those who practice medicine without the proper legal qualifications. In view of the fact that we felt this moral obligation resting upon us, we have done a great deal of work in excess of our legal duties. The secretary of the board has written to every superior court judge in the State asking them to particularly charge the grand juries in regard to this new law, and to request every solicitor-general to bring every offender against this law to justice. The secretary has given a great deal of time and labor to the work of getting up a list of the physicians in the State, with their postoffice address, the year in which they registered with the county clerks in accordance with the old law regulating the practice before the enactment of the new law. This was a tedious and laborious piece of work we imposed upon our secretary, but we considered it necessary before taking other steps contemplated, by which we hope to help our professional brethren to weed out the pretenders and quacks in the profession in this State. All of this and other work looking to the good of our profession has been done by the board without the hope of reward or fear of punishment.

In conclusion, will say we have asked no advice of the editor of the *Clinic*—perhaps we should have done so—but as an excuse for not doing so, will quote from Gœthe, "He who asks advice shows himself limited; he who gives it, gives also proof that he is presumptuous."

It is said that the goojerat or maneless lion of South Africa has a claw in his tail with which he lashes himself into a fury before attacking his prey. Now we will ask of our critic that when he sees a quack walking the streets of Atlanta, not to lash himself into a bad humor with the tail and claw of malice and attack this "board of figure heads," but go ahead and get up evidence against the offender, present him to the grand jury, get a true bill against him, then turn him over to Austin & Park, our attorneys, who have prosecuted offenders for us before. Then, we promise that we will not "hold up our hands in holy horror because there is no appropriation from the State" to pay Austin & Park, but we will pay them from our own pocket-books, as we have done heretofore.

E. R. ANTHONY, M.D.,
President of Board of Medical Examiners.

